# A study on the effect of remimazolam on cellular immune function during the perioperative period in older adults who are having laparoscopic surgery for colorectal cancer

**DOI:** 10.3389/fonc.2026.1827157

**Published:** 2026-08-03

**Authors:** Jiafang Wang, Lan Li, Zhong Qi, Zhijun Chen

**Affiliations:** 1 Department of Anesthesiology, Wuhan No.1 Hospital, Wuhan, China; 2 Department of Anesthesiology, Wuhan Wuchang Hospital, Wuhan, China

**Keywords:** elderly patients, immune function, laparoscopic radical resection of colorectal cancer, NK cells, propofol, remimazolam

## Abstract

**Objective:**

To investigate the effect of remimazolam on perioperative cellular immune function in elderly patients undergoing laparoscopic colorectal cancer surgery.

**Methods:**

Sixty elderly patients were randomly assigned to receive either propofol (Group P, n=30) or remimazolam (Group R, n=30) for anesthesia induction and maintenance. Hemodynamic parameters, Bispectral Index (BIS), emergence time, vasopressor requirement, and adverse reactions were recorded. T lymphocyte subsets (CD3^+^, CD4^+^, CD8^+^) and NK cells were measured by flow cytometry at designated timepoints (T0: pre-induction; T3: 60 minutes after establishing pneumoperitoneum; T5: 5min post-extubation; T6: 24h postoperatively), and the CD4^+^/CD8^+^ ratio was calculated.

**Results:**

Mean arterial pressure (MAP) at T5 was significantly higher in Group R compared to Group P (P<0.05). Group R had a significantly lower intraoperative vasopressor usage rate (P<0.05) but a longer emergence time (P<0.05). CD4^+^ cells and the CD4^+^/CD8^+^ ratio at T5 were significantly lower in Group R than in Group P (P<0.05). No significant differences were observed in other T-cell subsets, NK cells at other time points, intraoperative analgesic consumption, or the incidence of adverse reactions.

**Conclusion:**

No statistically significant difference was detected between remimazolam and propofol in the primary endpoint (NK cell percentage at 24 hours postoperatively); however, this superiority-designed study was not powered to demonstrate equivalence. Remimazolam reduced intraoperative vasopressor requirement but may prolong emergence time, with a temporary decrease in CD4^+^ cells and CD4^+^/CD8^+^ ratio at 5 minutes after extubation.

## 1 Introduction

The impact of anesthesia on patients’ perioperative immune function is gradually becoming an important focus in modern anesthesiology research. Existing evidence suggests that anesthetic agents can affect the tumor microenvironment by regulating immune cell function and inflammatory factors, thereby affecting the dissemination and metastasis of tumor cells (1–3). Propofol, a commonly used intravenous anesthetic in clinical practice, has some research backing its potential anti-tumor effects (such as inhibiting angiogenesis and regulating immune cell function) (4); however, its strong cardiovascular suppression (especially adverse events like hypotension and bradycardia that are common among older adults) limits its clinical application (5). Remimazolam is a novel ultra-short-acting benzodiazepine that has become more widely used in general anesthesia recently due to its rapid onset, stable metabolism, and minimal hemodynamic effects (6–8). However, research on the impact of remimazolam on perioperative immune function in cancer patients remains relatively limited. This study aims to investigate the effect of remimazolam on perioperative cellular immune function in elderly patients undergoing laparoscopic radical resection of colorectal cancer, with the goal of providing evidence-based insights for optimizing anesthesia protocols in oncologic surgery.

## 2 Materials and methods

### 2.1 General information

This study was approved by the hospital ethics committee of Wuhan No.1 Hospital (Kelun [2024] No. 33), and patients or their families signed informed consent forms. Elderly patients (age ≥ 65 years) scheduled for laparoscopic radical resection of colorectal cancer from July to December 2024 were selected, of any gender, with ASA grades I to III and BMI of 18 to 24 kg/m². Exclusion criteria: ① History of mental illness, severe depression, or long-term psychiatric medication use; ② Preoperative severe heart, brain, liver, kidney, endocrine diseases, or immune system diseases; ③ History of radiotherapy, chemotherapy, hormone therapy, or immunotherapy; ④ Presence of severe infections; ⑤ Preoperative pulmonary infections or respiratory failure; ⑥ Allergies to benzodiazepines and other anesthetics; ⑦ Long-term use of anticoagulants preoperatively. Patients were randomly divided into two groups using a digital table method: the Propofol group (P group) and the Remimazolam group (R group). The random allocation sequence was generated before enrollment by an investigator who was not involved in anesthesia management, postoperative follow-up, or statistical analysis. Group assignments were placed in sequentially numbered, sealed opaque envelopes and opened only after eligibility confirmation and informed consent, thereby maintaining allocation concealment before group assignment. Perioperative variables including operation duration, blood loss, blood transfusion, and crystalloid/colloid administration were recorded and assessed for intergroup balance. This study was not prospectively registered in a clinical trial registry. This was an open-label study. Outcome assessors responsible for postoperative follow-up and laboratory personnel measuring immune-cell subsets were blinded to group allocation; data analysts were also blinded before completion of the primary analysis. Anesthesia providers could not be blinded because remimazolam and propofol differ in preparation and administration characteristics.

### 2.2 Anesthesia methods

All patients fasted for 6 hours and refrained from drinking for 2 hours before surgery. Once in the operating room, we routinely monitored Heart rate (HR), electrocardiogram (ECG), blood pressure (BP), pulse saturation of pulse oximetry (SpO2), and BIS. Both groups underwent rapid intravenous induction; the P group received intravenous sufentanil 0.3 µg/kg (Yichang Renfu Pharmaceutical Co., Ltd., batch number: AB40401711), cisatracurium 0.15 mg/kg (Jiangsu Hengrui Medicine Co., Ltd., batch number: 240621XA), and propofol 1–2 mg/kg (Beijing Fresenius Kabi Pharmaceutical Co., Ltd., batch number: 16TC0414). The R group received intravenous sufentanil 0.3 µg/kg, cisatracurium 0.15 mg/kg, and remimazolam 0.2–0.3 mg/kg (Jiangsu Hengrui Medicine Co., Ltd., batch number: 230411AU). Once BIS values were ≤60 and muscle relaxation was satisfactory, tracheal intubation and mechanical ventilation were performed. Anesthesia maintenance: The P group received intravenous infusion of propofol 4–6 mg·kg^4^¹·h^4^¹ and remifentanil 0.1–0.3 µg·kg^4^¹·min^4^¹ (Yichang Renfu Pharmaceutical Co., Ltd., batch number: 20A11201); the R group received intravenous infusion of remimazolam 0.5–1.5 mg·kg^4^¹·h^4^¹ and remifentanil 0.1–0.3 µg·kg^4^¹·min^4^¹. We kept BIS values between 40 and 60, with intermittent additional doses of cisatracurium 0.05 mg/kg to maintain appropriate muscle relaxation. Throughout the surgery, efforts were made to maintain hemodynamic stability; atropine 0.01 mg/kg was administered for bradycardia (HR<50beats/min), and ephedrine 0.1–0.2 mg/kg was given for hypotension (mean arterial pressure (MAP) reduction exceeding 20% from baseline). After surgery, infusion of remimazolam or propofol and remifentanil was stopped, and the tracheal tube was removed once the patient was awake and had resumed spontaneous breathing. Anesthesia depth was strictly controlled with BIS maintained at 40–60 in both groups to ensure comparable hypnotic effect. No sedative premedication was routinely administered before entering the operating room. Apart from the assigned hypnotic agent, the induction and maintenance protocols were kept identical between groups, including the same sufentanil, cisatracurium, and remifentanil regimens, BIS target range, ventilation strategy, and criteria for vasoactive-drug administration. No neuraxial anesthesia, peripheral nerve block, or other regional anesthesia technique was used in either group.

### 2.3 Observation indicators

Primary endpoint: NK cell percentage at 24 hours postoperatively (T6). Secondary endpoints: perioperative hemodynamics, BIS, vasopressor requirement, emergence time, T lymphocyte subsets (CD3+, CD4+, CD8+), CD4+/CD8+ ratio, intraoperative analgesic consumption, and adverse reactions. Recorded HR, MAP, and pulse SpO2 for both groups at these time points: before anesthesia induction (T0), 1 minute after induction (T1), 5 minutes after pneumoperitoneum (T2), 60 minutes after establishing pneumoperitoneum (T3), at the end of surgery (T4), 5 minutes after extubation (T5), and 24 hours postoperatively (T6). BIS values were recorded from T0 to T5. The anesthesia duration, surgery duration, awakening time, intraoperative opioid analgesic dosage, vasoactive drugs usage rate, and incidence of postoperative adverse reactions were documented. At T0, T3, T5, and T6, we drew 5 ml of peripheral venous blood to measure T cell subpopulations (CD3+, CD4+, CD8+) and natural killer (NK) cells using the Gallios/Navios flow cytometer from Beckman Coulter, USA, and the CD4+/CD8+ ratio was calculated. To minimize research bias, all surgeries were performed by the same surgical team, the same anesthesia team managed the experimental drugs and anesthesia, and researchers were responsible for data collection and postoperative follow-up. Postoperative analgesia and supportive medications were standardized across groups. Patients received the same institutional postoperative analgesic regimen, including identical patient-controlled intravenous analgesia settings when PCIA was used, and adjunct analgesics, antiemetics, and other perioperative medications were administered according to the same departmental criteria in both groups.

### 2.4 Statistical methods

We analyzed the data using SPSS 19.0 statistical software. The primary observation indicator was NK cells at 24 hours postoperatively. According to preliminary results, NK cells in the P group at 24 hours postoperatively were 19.2% ± 4.2%, and in the R group, they were 22.6% ± 4.5%. Setting α = 0.05, 1-β = 0.8, and assuming a dropout rate of 20%, a total of 33 patients were planned for each group. Normally distributed measurement data were expressed as mean ± standard deviation (± s). The Shapiro-Wilk test was used to determine the normality of distribution, and Levene’s test was used for homogeneity of variance. Independent samples t-test was used for inter-group comparisons, and repeated measures ANOVA was used for intra-group comparisons. Multivariate analysis was performed to adjust for operation duration, blood loss, fluid administration, and other perioperative confounding factors to ensure the robustness of the results. Count data were expressed as the number of cases (%), and the comparison between count data was conducted using the χ2 test. A P value <0.05 was considered statistically significant. Benjamini-Hochberg (BH) false discovery rate correction was applied to account for multiple comparisons across immune parameters and time points to control Type I error. Because the immune-cell measurements at multiple time points were secondary outcomes, both unadjusted P values and the Benjamini-Hochberg adjusted interpretation were considered when evaluating these results, and secondary immune findings were interpreted as exploratory rather than definitive evidence of superiority.

## 3 Results

### 3.1 Comparison of general conditions between two groups

A total of 66 patients were included in this study, of which 3 patients from both the P group and the R group were excluded due to conversion to open surgery during the procedure, and there were no cases lost to follow-up. Ultimately, 60 patients were included in the analysis. There were no significant differences in age, gender, BMI, ASA classification, surgical time, anesthesia time, blood loss, blood transfusion, or crystalloid/colloid administration between the two groups (P > 0.05). The recovery time in the R group was significantly longer than in the P group, with a significant difference (P < 0.05), as shown in **Table 1**.

**Table 1:** Comparison of clinical characteristics between the two groups (*±s*).

Metrics	P	R	*t*/*χ^2^*	*P*
Age (years)	69.67 ± 407	71.80 ± 4.05	1.845	0.070
Male/Female	18/12	12/16	1.071	0.438
BMI (kg/m^2^)	22.31 ± 3.11	23.47 ± 3.05	1.454	0.151
ASA physical status (II/III)	8/22	5/25	0.884	0.532
Operation time (min)	240.17 ± 87.23	203.70 ± 48.41	-2.002	0.051
Anesthesia time (min)	312.50 ± 83.12	306.70 ± 54.38	-0.320	0.750
Emergence time (min)	14.47 ± 5.62	28.97 ± 12.62	5.750	<0.001

### 3.2 Comparison of MAP, HR, SpO_2_, and BIS values during the perioperative period in the two patient groups

Compared to T0, the MAP of both groups significantly decreased at T1, T3, and T4 (P<0.05). Additionally, the HR and BIS of both groups significantly decreased at T1, T2, T3, and T4 (P<0.05). In comparison to group P, the BIS in group R significantly increased at T1, and the MAP in group R significantly increased at T5 (P<0.05). No statistically significant difference in SpO_2_ was found between the two groups at different time points (P>0.05) (**Table 2**).

**Table 2:** Comparison of MAP、HR、SPO_2_ and BIS between the two groups (*±s*).

Metrics	Group	n	T0	T1	T2	T3	T4	T5	T6
MAP (mmHg)	P	30	89.4 ± 10.8	74.2 ± 11.3^*^	85.5 ± 12.7	76.2 ± 10.5^*^	81.1± 14.2^*^	84.3± 9.3	84.4 ± 9.3
	R	30	93.3 ± 10.2	77.6 ± 13.4^*^	86.5 ± 12.5	80.7 ± 10.1^*^	82.6 ± 14.0^*^	97.4± 13.4^a^	85.7 ± 11.9
HR times/min	P	30	71.77 ± 9.00	66.33 ± 10.88^*^	60.03 ± 8.54^*^	58.77 ± 7.50^*^	57.83 ± 10.27^*^	68.13 ± 10.97	68.67 ± 8.56
	R	30	74.73 ± 11.61	71.23 ± 14.50^*^	61.77 ± 10.88^*^	61.57 ± 12.29^*^	62.57 ± 12.92^*^	69.57 ± 12.34^*^	73.33 ± 9.96
SpO_2_(%)	P	30	95.5(93.0~99.0)	100.0(99.0~100.0)	100.0(99.0~100.0)	100.0(99.0~100.0)	100.0(99.0~100.0)	99.0(96.0~100.0)	95.0(92.0~100.0)
	R	30	96.0(94.0~99.0)	100.0(99.0~100.0)	100.0(98.0~100.0)	100.0(99.0~100.0)	100.0(98.0~100.0)	98.5(97.0~100.0)	95.5(93.0~100.0)
BIS	P	30	93.37 ± 4.26	43.70 ± 5.21^*^	46.87 ± 8.34^*^	46.87 ± 6.90^*^	64.47 ± 11.82^*^	87.07 ± 9.65^*^	–
	R	30	93.13 ± 10.0	50.90 ± 10.78^*a^	48.43 ± 7.21^*^	48.60 ± 9.02^*^	66.93 ± 11.71^*^	89.53 ± 5.12	–

Compared with T0, *P<0.05; compared to group P, aP<0.05.

### 3.3 Comparison of medication during surgery between the two groups

**Figure 1:**
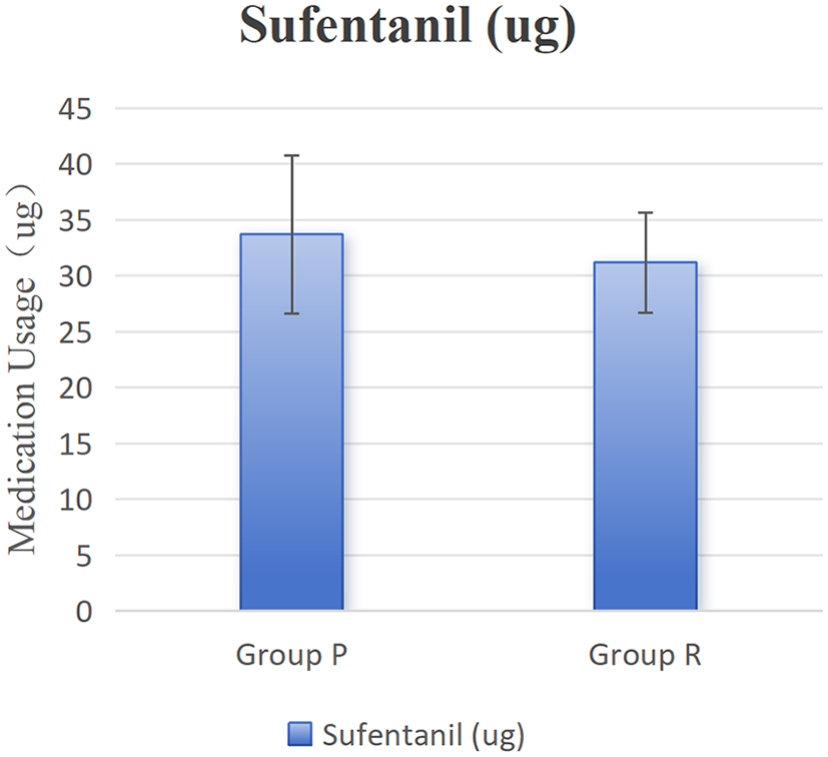
Intraoperative sufentanil dosage in group P and group R (mean ± SD, error bars represent standard deviation).

**Figure 2:**
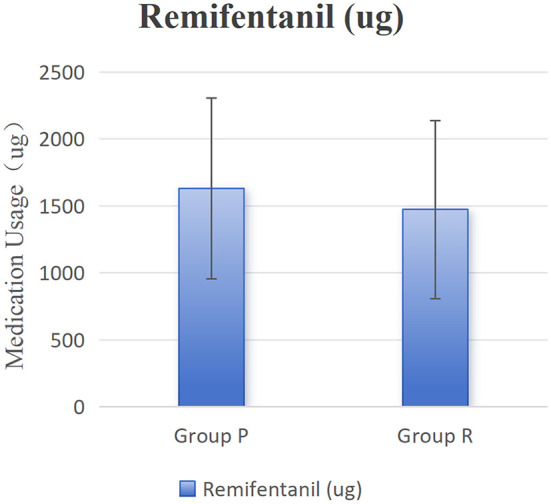
Intraoperative remifentanil dosage in group P and group R (mean ± SD, error bars represent standard deviation).

**Figure 3:**
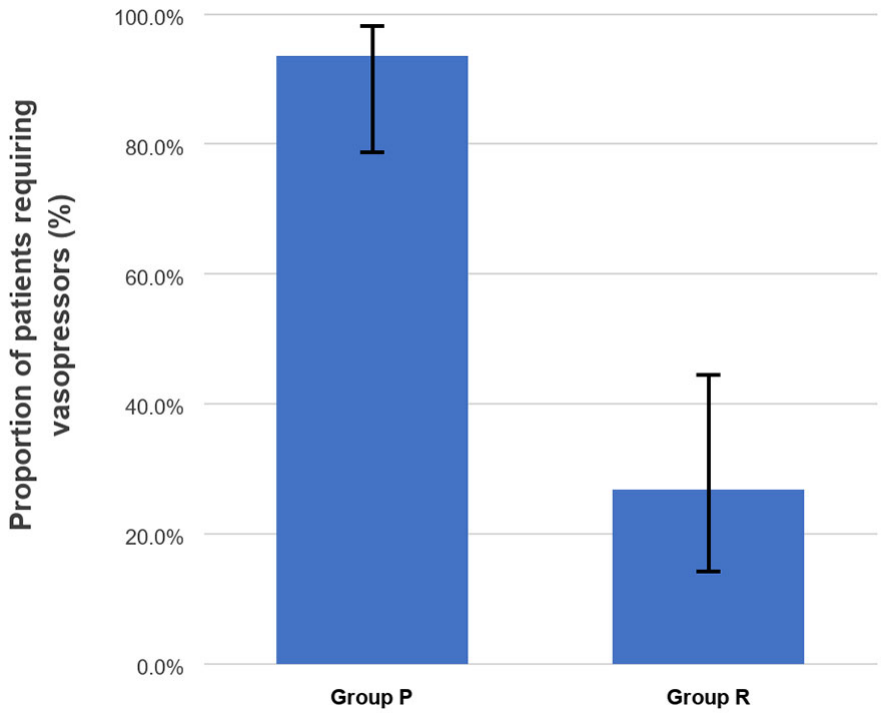
Comparison of intraoperative vasopressor requirement between the Propofol group (Group P) and the Remimazolam group (Group R). Note: Data are presented as proportions with 95% confidence intervals (error bars, calculated using the Wilson method). The risk difference (RD) between Group R and Group P was -66.7% (95% CI: -84.3% to -49.0%; P < 0.001), indicating a significantly lower requirement for vasopressors in the Remimazolam group.

The rate of vasoactive drugs in group R during the operation was significantly lower than in group P (P<0.05). There was no statistically significant difference in the dosage of analgesics intraoperative between the two groups (P>0.05). As shown in **Table 3**; **Figures 1**–**3**.

**Table 3:** Comparison of intraoperative medication use between the two groups of patients (*±s*).

Intraoperative medication	P	R	*t/χ^2^*	*P*
Sufentanil (μg)	33.70 ± 7.07	31.17±4.49	-1.657	0.104
Remifentanil (μg)	1630.59±676.02	1472.93±663.94	0.911	0.366
Pressor drugs (%)	28(77.8)	8(22.2)^a^	27.778	<0.001

Compared to group P, aP<0.05.

### 3.4 Comparison of immune indicators at different time points between two groups of patients

**Figure 4:**
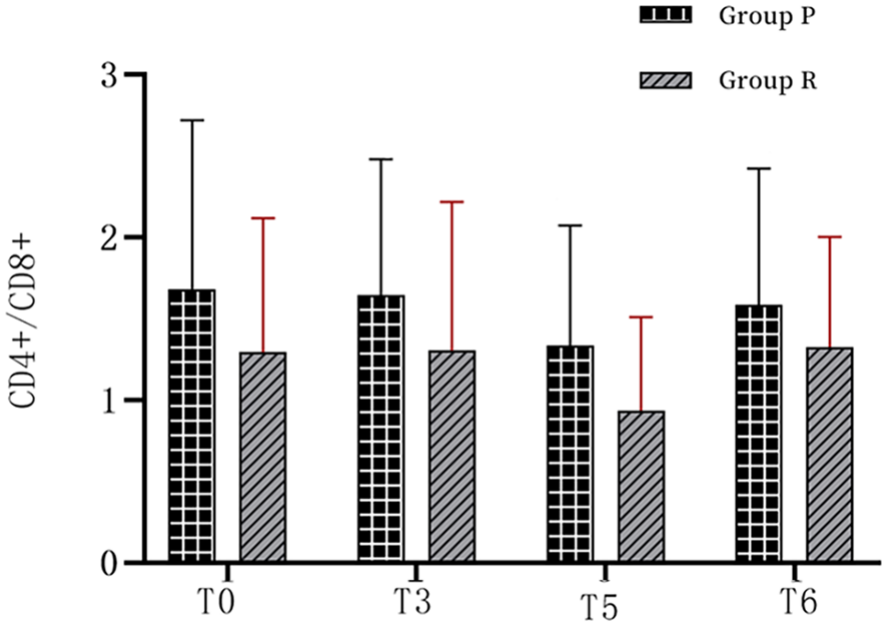
Comparison of immune indicators at different time points between the two groups.

**Figures 5:**
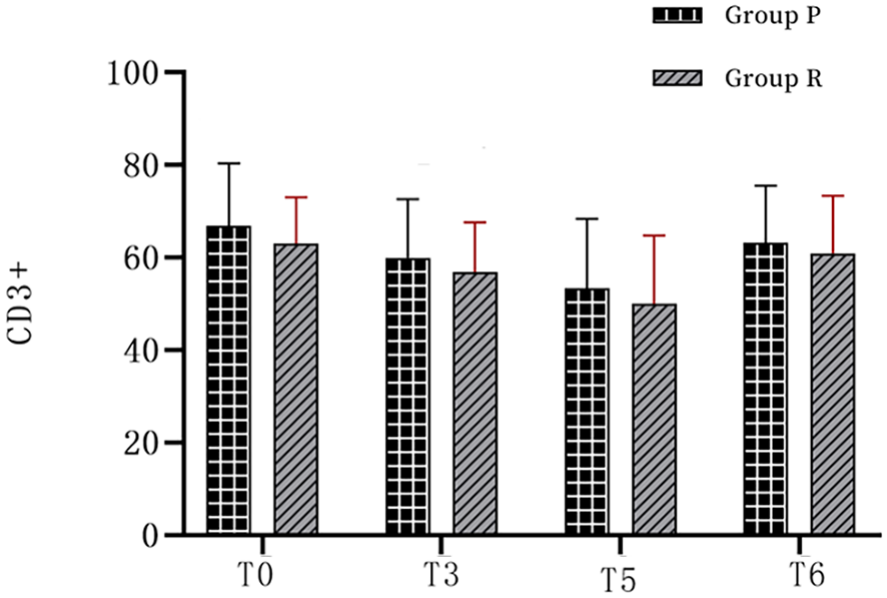
Comparison of immune indicators at different time points between the two groups.

**Figure 6:**
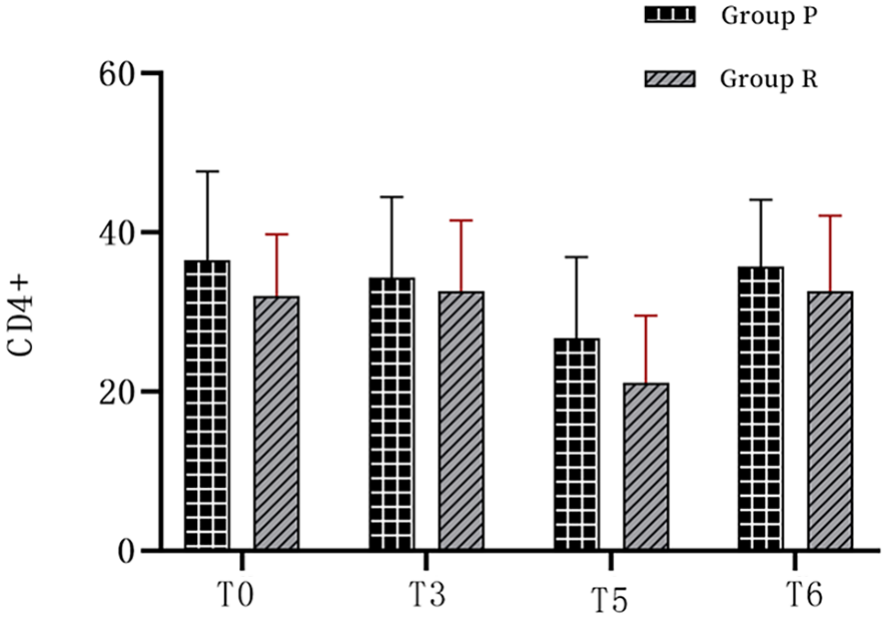
Comparison of immune indicators at different time points between the two groups.

**Figure 7:**
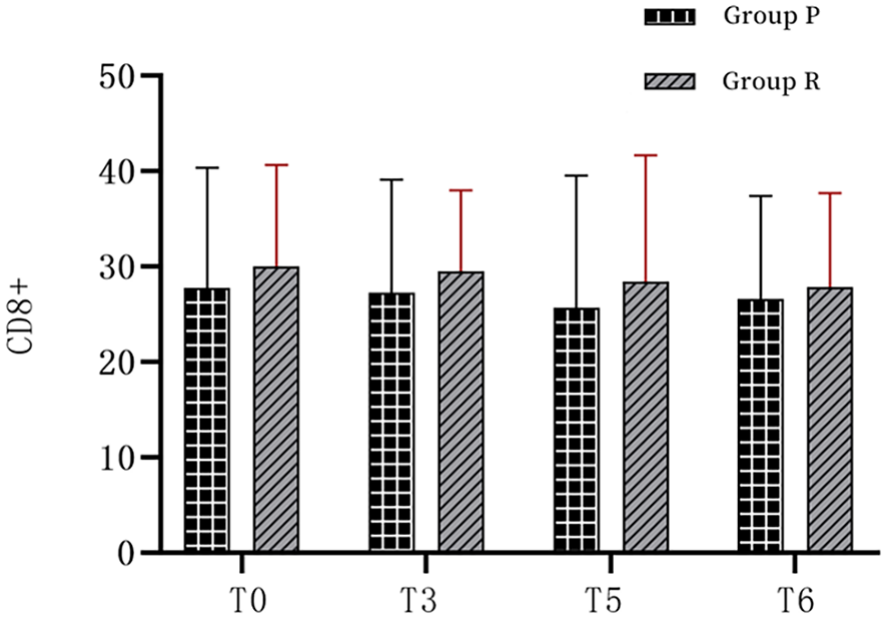
Comparison of immune indicators at different time points between the two groups.

**Figure 8:**
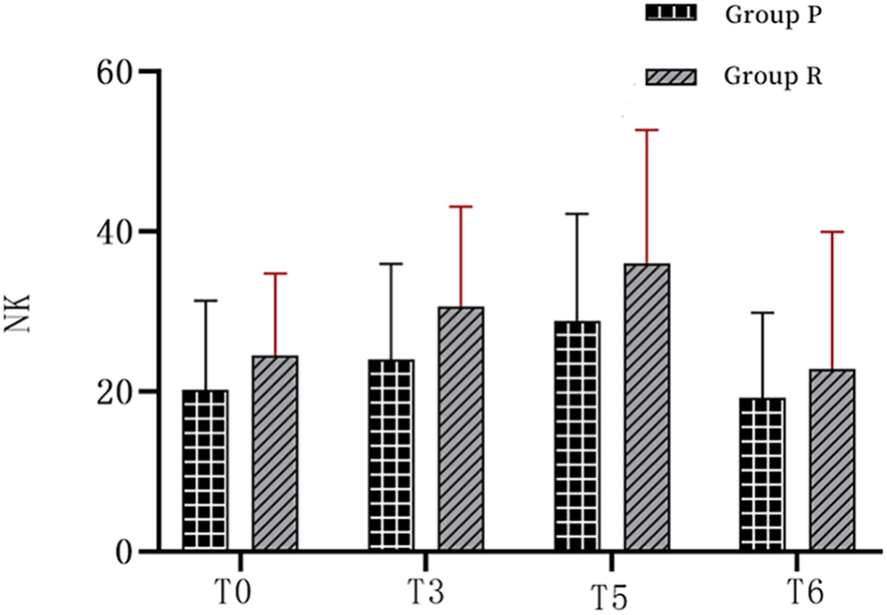
Comparison of immune indicators at different time points between the two groups.

No significant between-group differences were detected in CD3+, CD4+, CD8+, NK cells, or CD4+/CD8+ ratio at baseline T0 (all P> 0.05). The minor visual difference in the CD4+/CD8+ ratio at T0 in **Figure 4** was caused by graph scaling and did not reflect a statistically significant baseline imbalance. Accordingly, baseline adjustment using analysis of covariance was not performed, and conventional between-group comparisons were applied for all analyses. No significant difference was observed in the primary endpoint (NK cell percentage at 24 hours postoperatively, T6) between the two groups (P > 0.05). For intraoperative vasopressor requirement, the risk difference between Group R and Group P was -66.7% (95% CI: -84.3% to -49.0%), indicating a significantly lower vasopressor requirement in Group R. When compared to T0, the number of CD3+ cells in the P group significantly decreased at T3, T5, and T6, whereas the CD4+ and CD8+ cells, along with the CD4+/CD8+ ratio, significantly decreased at T5. NK cells were significantly elevated at T3 and T5. In the R group, the number of CD3+ cells significantly decreased at T3 and T5, the number of CD4+ cells and the CD4+/CD8+ ratio significantly decreased at T5, the number of CD8+ cells significantly decreased at T6, and NK cells were significantly elevated at T3 and T5 (P < 0.05). The R group exhibited a significant decrease in CD4+ cells and the CD4+/CD8+ ratio at T5 compared to the P group (P < 0.05). No statistically significant differences were found in CD3+, CD4+, CD8+, NK cells, and the CD4+/CD8+ ratio between the two groups at T0, T3, and T6, and no statistical differences were observed in CD3+, CD8+, and NK cells at T5 (P > 0.05) (refer to **Table 4**; **Figure 4**–**8**). All statistically significant differences reported remained valid after Benjamini-Hochberg correction for multiple comparisons. The significant between-group differences in CD4+ cells and the CD4+/CD8+ ratio at T5 remained significant after Benjamini-Hochberg false discovery rate correction, whereas no additional between-group immune differences emerged after adjustment.

**Table 4:** Comparison of cellular immunity index between the two groups (*±s*).

Immune markers	Group	T0	T3	T5	T6
CD3^+^(%)	P	66.87 ± 13.49	60.00 ± 12.59^*^	53.37±15.01^*^	63.18±12.34^*^
	R	63.04 ± 9.96	56.86 ± 10.73^*^	50.06±14.72^*^	60.96±12.41
CD4^+^(%)	P	36.59 ± 11.08	34.32 ± 10.16	26.69±10.24^*^	35.70±8.44
	R	32.04 ± 7.72	32.62 ± 8.88	21.14±8.43^*a^	32.61±9.52
CD8^+^(%)	P	27.76 ± 12.62	27.29 ± 11.86	25.69±13.85^*^	26.60±10.84
	R	30.06 ± 10.64	29.54 ± 8.48	28.43±13.26	27.88±9.84^*^
NK(%)	P	20.25 ± 11.11	24.04 ± 11.91^*^	28.86±13.35^*^	19.20±10.70
	R	24.47 ± 10.30	30.70 ± 12.43^*^	36.07±16.67^*^	22.82±17.16
CD4^+^/CD8^+^	P	1.68 ± 1.04	1.65 ± 0.83	1.33±0.74^*^	1.59±0.84
	R	1.29 ± 0.82	1.30 ± 0.92	0.94±0.58^*a^	1.33±0.68

Compared with T0, *P<0.05; compared to group P, aP<0.05. P > 0.05 for all between-group comparisons at T0.

### 3.5 Comparison of the incidence of postoperative side effects between the two groups

The side effects included agitation in the recovery phase, nausea and vomiting after surgery, drowsiness, and itching. There was no significant difference between the two groups (P > 0.05) (**Table 5**).

**Table 5:** Comparison of the incidence of postoperative adverse reactions between the two groups of patients n(%).

Complication [n (%)]	P group	R group	*χ^2^*	*P*
Emergence agitation (EA)	3(10.0)	2(6.7)	0.218	0.669
Postoperative nausea and vomiting (PONV)	5(16.7)	8(26.7)	0.884	0.347
Somnolence	0(0)	4(13.3)	3.281	0.113
Pruritus	0(0)	1(3.3)	0.254	1.000

Comparison of postoperative complications between the two groups n(%).

## 4 Discussion

Colorectal cancer is now the third most common cancer globally and is also one of the most prevalent cancers among elderly patients, imposing a substantial economic burden on patients and society (9). Currently, surgical resection remains one of the most effective methods for treating colorectal cancer and metastatic lymph nodes. However, there residual tumor tissue may remain after surgical resection, and some tiny metastases can spread through the lymphatic and blood systems during surgery. Also, surgical trauma can trigger the hypothalamic-pituitary-adrenal axis and the sympathetic nervous system, leading to more pro-inflammatory cytokines being released, suppressing cell-mediated immunity, and increasing the risk of tumor metastasis and recurrence (10). The anesthetics, anesthesia techniques, and related perioperative factors can have a positive or negative impact on the patient’s immune function. This, in turn, might affect the long-term prognosis for cancer patients (11–13). So, minimizing the impact of anesthesia on perioperative immune function in cancer patients is an important focus for anesthesiologists. The interaction between anesthesia and immunity has also become a hot research topic in recent years, focusing mainly on the effects of anesthetic drugs on perioperative immune function and their impact on tumor prognosis (14).

Propofol is now the most commonly used intravenous general anesthetic. It boosts the activity of cytotoxic T cells, reduces the production of pro-inflammatory cytokines, and alleviates the immunosuppressive effects of surgery and anesthesia (15, 16). However, propofol can significantly suppress the circulatory system, particularly in elderly or frail patients, which can lead to adverse reactions such as hypotension and bradycardia, increasing the likelihood of ischemic cardiovascular events during the perioperative period and potentially affecting patient prognosis (17). Remimazolam is a new ultra-short-acting benzodiazepine that acts on the gamma-aminobutyric acid type A (GABA) receptors in the central nervous system, hyperpolarizing neuronal cell membranes, thereby inhibiting neuronal activity and causing sedation. It has the advantages of rapid onset, quick recovery, independence from hepatic and renal metabolism, and minimal impact on hemodynamics, and is now widely used for the induction and maintenance of general anesthesia. Current research shows that remimazolam has significant advantages in patients with unstable circulatory function, impaired liver and kidney function, and elderly frailty, Remimazolam offers distinct benefits, since it mildly inhibits BP and HR levels, and significantly reduces the requirement for vasoactive drugs (18). In the results of this study, although both groups of patients saw significant drops in BP and HR during anesthesia, only the MAP in the remimazolam group was higher than that in the propofol group after extubation. However, the use of vasoactive drugs in the remimazolam group was significantly lower than in the propofol group. Analyzing the possible reasons, during the anesthesia of elderly patients, anesthesiologists often pay more attention to the patients’ circulatory status and tend to intervene promptly before a trend of BP decline occurs to ensure patient safety and avoid severe hypotension. This study also shows that, compared to propofol, remimazolam causes less circulatory suppression in elderly patients, which can reduce the use of vasoactive drugs and maintain stable circulatory function, potentially benefiting patient prognosis. The results of this study indicate that the awakening time of patients in the remimazolam group was significantly prolonged compared to the propofol group, which is consistent with some current research findings (19, 20). Analyzing the possible reasons, elderly patients experience a gradual decline in drug metabolism and brain function, often accompanied by varying degrees of hypoalbuminemia, which may increase sensitivity to benzodiazepines (21). Additionally, the dose-response relationship of remimazolam with BIS is not as pronounced as that of propofol (22). In this study, to avoid awareness during surgery, we kept the BIS between 40 and 60. The BIS levels were comparable between the two groups. Therefore, it is possible that the dose of remimazolam used in this study for elderly patients was relatively high, which could also explain the prolonged awakening time. Caution is therefore warranted regarding the risk of prolonged emergence when remimazolam is used over extended periods in elderly patients. Meanwhile, a clinical trade-off exists in this study: remimazolam offers more stable hemodynamics and significantly reduces intraoperative vasopressor requirement, which is particularly favorable for elderly patients with cardiovascular fragility, but it also leads to prolonged emergence time. This balance between circulatory safety and recovery speed should be fully considered in clinical anesthesia decision-making. In terms of the incidence of adverse reactions such as agitation during the awakening period, postoperative nausea and vomiting, drowsiness, and itching, this study did not demonstrate a clear advantage of remimazolam.

T lymphocytes mediate cellular immunity and play an extremely important role in the body’s anti-tumor immunity. CD3+ T cells indicate the overall level of cellular immunity. CD4+ T cells are helper T cells which boost the anti-tumor immune response by activating CD8+ T cells, promoting antigen presentation, enhancing antibody responses, and maintaining immune memory (23). CD8+ T cells, as core anti-tumor effector cells, can directly attack tumor cells and secrete cytokines such as interferon-gamma (IFN-γ) to inhibit tumor growth and metastasis (24). The CD4+/CD8+ ratio is a key indicator for assessing immune balance, and a decreased ratio indicates that cellular immunity might be suppressed (25). NK cells, as key players in innate immunity, can directly kill tumor cells and secrete cytokines to activate adaptive immunity, playing a crucial role in tumor immune surveillance and immunotherapy (26). Current research has shown notable differences in how different anesthetics regulate perioperative cellular immune function in surgical patients. In this study, no statistically significant between-group difference was detected in the primary endpoint, NK cell percentage at 24 hours postoperatively; nevertheless, the trial was not designed or powered to establish equivalence between remimazolam and propofol. However, the remimazolam group showed a more significant decrease in CD4+ immune cells and the CD4+/CD8+ ratio at the time point T5. The transient difference in immune indicators at T5 was attributed to the pharmacological characteristics of remimazolam, not to differences in anesthesia depth or unbalanced confounding factors. This result might be linked to the immunomodulatory characteristics of benzodiazepines. Studies have shown that the GABA signaling pathway is not confined to the central nervous system but is also widely distributed in immune cells (such as T cells and macrophages). Activation of GABA receptors can inhibit T cell proliferation and the secretion of Th1-type cytokines (such as IFN-γ), which can weaken the cellular immune response (27). Current research findings regarding the changes in NK cells during surgery are inconsistent. Wu et al. reported that in patients undergoing laparoscopic colorectal cancer surgery, NK cell ratios did not show significant changes in the remimazolam group during surgery and at 24 hours postoperatively. (28). However, Wu et al. (29) found that NK activity increased rapidly within 24 hours postoperatively. In this study, NK cell counts increased significantly in both groups at T3 and T5. Possible reasons for this involve surgical stress and inflammatory mediators stimulating NK cell proliferation and activation, leading to a short-term transient increase in their numbers; the decrease in T cell subset numbers resulted in a relative increase in the NK cell ratio. However, NK cell function relies not just on their numbers but also on their cytotoxic activity. Recent studies have shown that NK cells are cells that utilize GABA. Activating GABA receptors in laboratory settings can inhibit the cytotoxic activity of NK cells. Therefore, the actual anti-tumor effects of remimazolam on NK cells warrant further investigation, as noted in a study (30). In addition, this study did not set postoperative infection rate, hospital length of stay, or quality of postoperative recovery as clinical endpoints, so the comprehensive clinical benefits of remimazolam could not be fully evaluated. These key clinical outcomes should be included in future research to further clarify its perioperative clinical value.

The limitations of this study are as follows: the actual counts of immune cells and the levels of cytokines (such as IL-6 and TNF-α) were not measured, which limits a full assessment of the patients’ immune status; the absence of long-term follow-up data also precludes a clear determination of the effect of remimazolam on tumor recurrence; the heterogeneity of the elderly population (such as comorbidities and nutritional status) may have biased the results. In addition, the lack of prospective clinical trial registration is also an important limitation of this study. And this was an open-label trial without blinding of anesthesia providers, which may introduce potential bias and is a methodological limitation. Future multicenter studies are needed to clarify how it affects the long-term prognosis for cancer patients. This study only measured the percentages of lymphocyte subsets without absolute counts or total lymphocyte counts. In addition, cytokine levels, proliferative activity, and cytotoxic function were not evaluated, which limits the comprehensive interpretation of immune status. Moreover, this study only included a single postoperative time point at 24 hours after surgery, without longer-term monitoring such as postoperative day 3 or day 7. Given that the perioperative immunosuppression in elderly patients may persist for several days, the current time point design can only reflect the acute early postoperative immune response. Long-term immune recovery and sustained drug-related immune changes could not be further evaluated. These limitations have been acknowledged and objectively discussed in the present study. Finally, although Benjamini-Hochberg false discovery rate correction was applied for multiple immune-marker and time-point comparisons, these analyses involved secondary endpoints in a modest single-center sample. Therefore, the significant T5 differences should be interpreted cautiously as exploratory findings that require confirmation in larger, prospectively registered, blinded multicenter trials with prespecified multiplicity control.

In summary, no statistically significant difference was detected between remimazolam and propofol in the primary endpoint (NK cell percentage at 24 hours postoperatively) in elderly patients undergoing laparoscopic radical colorectal cancer surgery; however, this study was not powered to demonstrate equivalence. Remimazolam reduces the usage rate of vasoactive drugs during surgery, but prolonged infusion may lead to extended awakening time, accompanied by a temporary decrease in CD4+ cells and CD4+/CD8+ ratio at extubation.

## Data Availability

The original contributions presented in the study are included in the article/supplementary material. Further inquiries can be directed to the corresponding author.
